# Emerging malaria in Indonesia: An overview of *Plasmodium knowlesi* infections

**DOI:** 10.1016/j.parepi.2024.e00405

**Published:** 2024-12-27

**Authors:** Nisa Fauziah, Karomahul Malaya Jati, Fedri Ruluwedrata Rinawan, Naufal Fakhri Nugraha, Bachti Alisjahbana, Jontari Hutagalung

**Affiliations:** a Division of Parasitology, Department of Biomedical Sciences, Faculty of Medicine, Universitas Padjadjaran, Jl. Prof. Eyckman No. 38, Sukajadi, Bandung 40161, West Java, Indonesia; b Laboratory of Parasitology, Faculty of Medicine, Universitas Padjadjaran, Jl. Prof. Eyckman No. 38, Sukajadi, Bandung 40161, West Java, Indonesia; c Postgraduate Program, Faculty of Medicine, Universitas Padjadjaran, Jl. Prof. Eyckman No. 38, Sukajadi, Bandung 40161, West Java, Indonesia; d Research Center for Care and Control of Infectious Disease, Universitas Padjadjaran, Jl. Prof. Eyckman No. 38, Sukajadi, Bandung 40161, West Java, Indonesia; e Graduate Medical Study Program, Faculty of Medicine, Universitas Padjadjaran, Jl. Ir. Soekarno KM. 21, Jatinangor, Sumedang 45363, West Java, Indonesia; f Department of Public Health, Faculty of Medicine, Universitas Padjadjaran, Jl. Ir. Soekarno KM. 21, Jatinangor, Sumedang 45363, West Java, Indonesia; g Center for Health System Study and Health Workforce Education Innovation, Faculty of Medicine, Universitas Padjadjaran, Jl. Prof. Eyckman No. 38, Sukajadi, Bandung 40161, Indonesia; h Indonesian Society for Remote Sensing Branch West Java, Gedung 2, Faculty of Fisheries and Marine Sciences, Universitas Padjadjaran, Jl. Ir. Soekarno KM. 21, Jatinangor, Sumedang 45363, Indonesia; i Universitas Padjadjaran Hospital, Jl. Ir. Soekarno KM. 21, Jatinangor, Sumedang 45363, West Java, Indonesia; j Department of Internal Medicine, Faculty of Medicine, Universitas Padjadjaran/Dr. Hasan Sadikin General Hospital, Jl. Prof. Eyckman No. 38, Sukajadi, Bandung 40161, West Java, Indonesia; k Laboratory of Parasitology, Center for Health Resilience System Policy and Health Resources, Health Policy and Development Agency, Jl. Percetakan Negara No. 29, Johar Baru, Central Jakarta 10560, DKI Jakarta, Indonesia

**Keywords:** Indonesia, Infection, Malaria, Overview, *Plasmodium knowlesi*

## Abstract

**Background:**

*Plasmodium knowlesi*, the fifth malaria-causing parasite species, is currently changing the landscape of the most dominant malaria-causing species in the Southeast Asia by becoming the emerging significant cause of malaria in the region, including in Indonesia. This study aimed to provide an overview of malaria caused by *P. knowlesi* in Indonesia.

**Methods:**

This study utilized secondary data from the Indonesian National Referral Malaria Laboratory from 2011 to 2020 for the analysis.

**Results:**

Analysis on 212 samples collected over ten years identified 66 (31.1 %) cases of *P. knowlesi* infection, with one (0.5 %) mixed infection of *P. knowlesi* and *P. vivax*. These cases were reported in seven provinces in Kalimantan and Sumatra islands. Males were 2.23 times more likely to be at risk for malaria compared to females, and this result was statistically significant (*p*-value = 0.037, 95 % CI: 0.84–5.91). There was no significant association between the risk of malaria and the age groups classified as non-productive and productive (*p*-value = 0.535, OR = 0.42, 95 % CI: 0.12–1.53). Individuals working outdoors were not significantly more protected compared to those working indoors (p-value of 0.116, OR = 0.15, 95 % CI: 0.02–1.49). The origin of the sample was found to be the most significant factor (p-value <0.001), with individuals from Kalimantan having the highest risk for malaria caused by *P. knowlesi* (OR = 3.97, 95 % CI: 2.10–7.49).

**Conclusions:**

Two major Indonesian islands of Sumatra and Kalimantan, which reported malaria cases during the period studied, exhibit a potential risk for *P. knowlesi* infections that is influenced by factors beyond natural hosts and vectors, such as sex, age, and occupation. Routine PCR examinations for suspected *P. knowlesi* infections are crucial for developing effective strategies to identify and control this simian malaria parasite.

## 1 Introduction

Malaria is an infectious disease caused by protozoan of the *Plasmodium* genus. Six species of this genus, *P. falciparum*, *P. vivax*, *P. malariae*, *P. knowlesi, P. ovalewallikeri,* and *P. ovalecurtisi* have been identified as the causes of malaria in humans *(*Snounou et al., *2024**)*. Various factors, including climate and environment, influence global malaria distribution (Cella et al., 2019; Okunlola and Oyeyemi, 2019), leading to a diversity in vectors and *Plasmodium* species that infect humans (Gunathilaka et al., 2015; Ageep et al., 2009). In Africa, *P. falciparum* is the leading cause of malaria (Guerra et al., 2008; World Health Organization, 2022). In contrast, *P. vivax* is the primary cause of malaria in the Middle East, Asia, and the Western Pacific (Wongsrichanalai et al., 2019; Al-Awadhi et al., 2021). Previous findings also indicated that within the Malaysian Peninsula, particularly in Sabah region of Malaysian Borneo, *P. knowlesi* is the indigenous malaria parasite with a high incidence (Yusof et al., 2016; Chin et al., 2021; Ahmed and Cox-Singh, 2015; Zaw and Lin, 2019a). Thus, *P. knowlesi* poses a growing public health concern in the Southeast Asia (Shearer et al., 2016), with infections primarily occurring in forested areas where the natural host, monkeys (*Macaca fascicularis*), interact with humans (Barber et al., 2017; Benavente et al., 2019).

*P. knowlesi* is the *Plasmodium* species capable of infecting humans and causing malaria (White, 2008). In 2022–2023, 120 malaria cases caused by *P. knowlesi* have been reported in Indonesia based on the results of microscopic and polymerase chain reaction (PCR) analyses (Singh et al., 2004). The recognition of *P. knowlesi* is crucial, given that this species can cause high parasitemia and the standard treatment guideline for each *Plasmodium* species is different (Cox-Singh et al., 2008; Millar and Cox-Singh, 2015). Furthermore, testing is a foundational element for implementing monitoring programs to track the spread of *P. knowlesi* infections.

A recent study shows that the epicentre of malaria cases that are caused by *P. knowlesi* infection is the Southeast Asia region. All countries in this region, except Timor Leste, have reported *P. knowlesi* malaria cases (Vythilingam et al., 2018). These countries include Cambodia (Yek et al., 2022), Vietnam, Laos (Pongvongsa et al., 2018), Thailand (Sugaram et al., 2021), Myanmar (Jiang et al., 2010), and the Philippines (Malijan et al., 2021). Malaysia, the country with the highest number of *P. knowlesi* infections (Zaw and Lin, 2019b), reported 8473 and 3524 malaria *P. knowlesi* cases in Sarawak (2011–2019) (Ooi et al., 2022) and Sabah (2015–2017) of Borneo (Cooper et al., 2020), respectively. In addition to Malaysia, other countries located in Borneo Island (Brunei and Indonesia) have also documented a significant number *P. knowlesi* cases (Jeyaprakasam et al., 2020). This statement is reinforced by the geographical presence of natural hosts and vectors of *P. knowlesi* within the specified area, including Indonesia (Jeyaprakasam et al., 2020; Bin Said et al., 2022).

The first reported malaria *P. knowlesi* case in Indonesia occurred in 2010 in South Kalimantan Province, involving a patient who had previously worked near a forested area (Figtree et al., 2010). In 2013–2014, three cases were reported in Borneo: one in South Kalimantan Province and two in Central Kalimantan Province (Ompusunggu et al., 2015). Aceh province reported 16 *P. knowlesi* cases in 2014 (Herdiana et al., 2018) while Jambi Province reported 34 *P. knowlesi* infections in 2015 (Salwati et al., 2017). A study in North Sumatra Province reported 377 *P. knowlesi* infections in 2015 (Lubis et al., 2017a), and another study in Aceh province reported 111 cases of *P. knowlesi* infection during the period of 2015–2018 (Zohra et al., 2019). Although several studies have reported *P. knowlesi* infections in Indonesia, significant gaps remain in understanding these cases, particularly regarding epidemiological distribution, control, and prevention. (World Health Organization Regional Office for Western Pacific, 2017). To effectively address the issue of *P. knowlesi* malaria, Indonesia needs to understand the case characteristics and distribution. However, as mentioned above, further studies on *P. knowlesi* malaria, particularly epidemiological studies, are still needed in Indonesia. This study aimed to provide an overview of malaria *P. knowlesi* cases based on national cross-checking data.

## 2 Methods

We analyzed secondary data from the national referral laboratory, i.e., the 2011–2020 national cross-checking data, of suspected *P. knowlesi* malaria cases. Variables studied were patient demographic characteristics, microscopic examination results, and PCR examination results.

### 2.1 National cross-checking data of *P. knowlesi* suspects

**Fig. 1:**
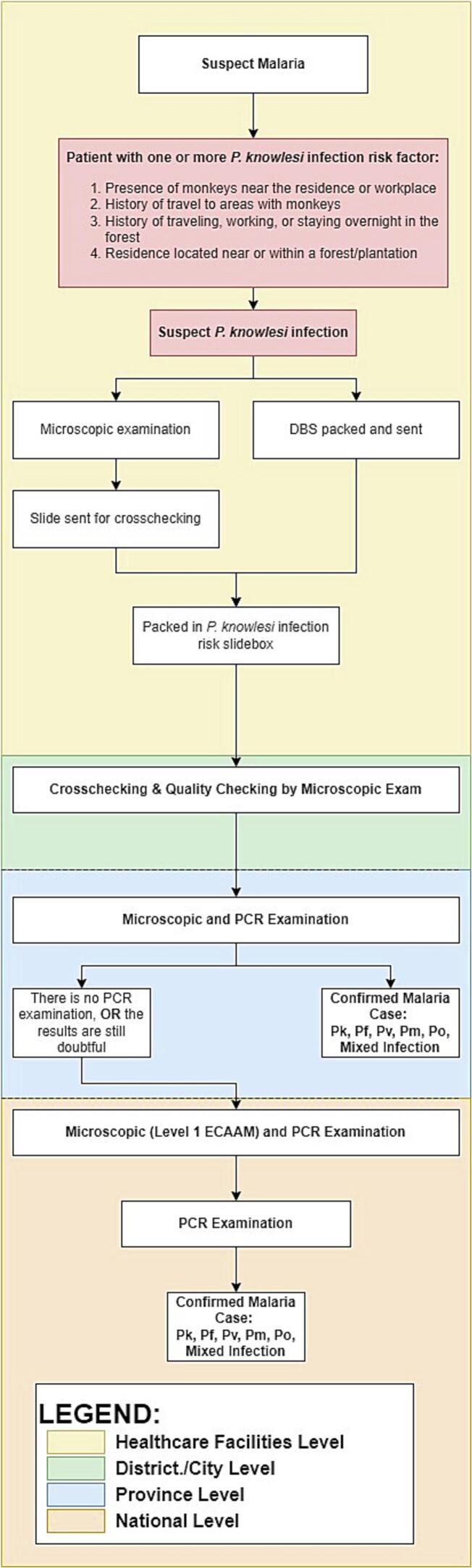
Malaria Laboratory Examination Network for *P. knowlesi* Suspects in Indonesia.

**Fig. 2:**
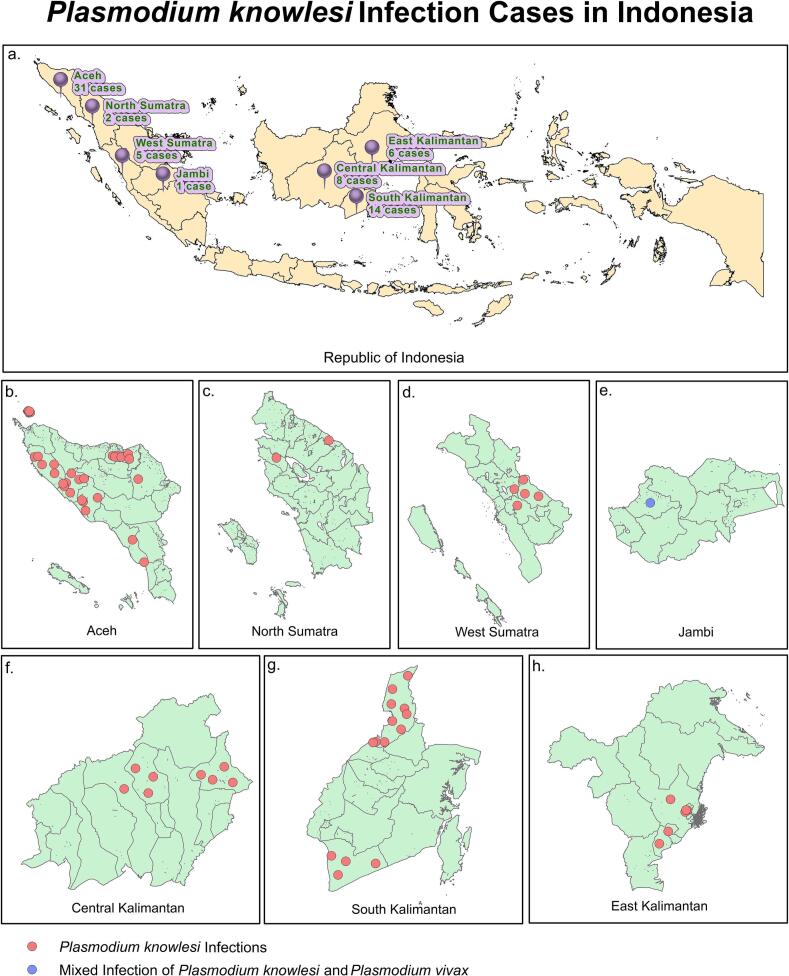
Geographic Distribution of Confirmed *P. knowlesi* Infections in Indonesia from 2011 to 2020 based on national cross-checking data. (a) provides an overview of *P. knowlesi* cases across all provinces in Indonesia; (b) through (h) offer a more detailed view of infection distribution within specific provinces of (b) Aceh, (c) North Sumatra, (d) West Sumatra Province, (e) Jambi, (f) Central Kalimantan, (g) South Kalimantan, (h) East Kalimantan.

**Fig. 3:**
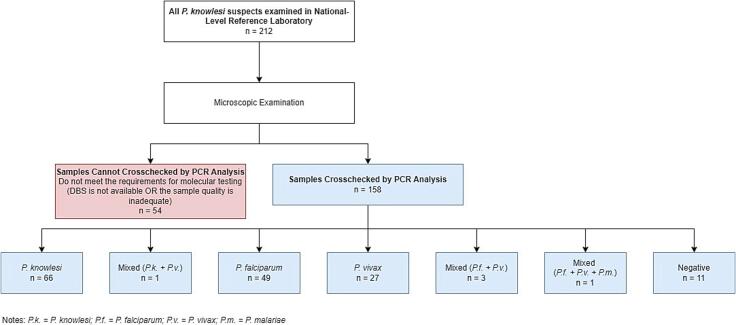
*P. knowlesi* Suspect Cross-Checking Examination Results from National-level Reference Laboratory.

Indonesia's laboratory network for malaria examinations spans from primary health services to the national level, encompassing four tiers: health care facility laboratories, district-level reference laboratories, province-level reference laboratories, and national-level reference laboratory (Fig. 1). This network is responsible for diagnosing and managing suspected malaria cases and designed to support malaria elimination programs and conduct periodic training for the lower laboratory levels. Each level of the malaria laboratory network has specific duties. The health care facility laboratory conducts microscopic examinations and provides rapid diagnostic test, followed by explanation and interpretation of the result. The district-level reference laboratory performs cross-checking examinations while the province-level reference laboratory conducts PCR examinations and cross-checking examinations if there are any discrepancies or discordance at lower levels. Only the national level reference laboratory has the capability for PCR examinations (Ministry of Health of the Republic of Indonesia, 2020). (See Fig. 2, Fig. 3.)

Unlike other Plasmodium species, PCR confirmation is mandatory for identifying *P. knowlesi*. Not all provincial laboratories can identify *P. knowlesi* at the molecular level. Therefore, samples from suspected *P. knowlesi* malaria cases that cannot be determined at the provincial level must be referred to the national reference laboratory for confirmation testing. In the national-level reference laboratory, all suspected *P. knowlesi* infections are initially examined microscopically, and then confirmed using the PCR testing. The PCR examination for *P. knowlesi* employs a single-step PCR targeting the Sr-RNA-SICAvar gene, which is aspecific schizont-infected cell agglutination variant antigen, with a product size of 228 bp (Lubis et al., 2017b). The Ministry of Health provides oversight for microscopists to control the quality of malaria diagnoses. While PCR confirmation is mandatory for *P. knowlesi,* diagnosis for other Plasmodium species can be done using the microscopy alone. Consequently, PCR testing is not routinely conducted unless *P. knowlesi* infection is suspected. However, with higher-level PCR confirmation, this tiered reference network ensures that all suspected *P. knowlesi* cases undergo the necessary molecular testing (Directorate General of Disease Prevention and Control M of H of TR of I, 2017). (Ministry of Health of the Republic of Indonesia, 2020)

### 2.2 Statistical analysis

Data were analyzed using STATA SE version 17. Associations between categorical variables were assessed through a chi-square test to obtain *P*-values, while logistic regression was used to calculate odds ratios, determining the strength and direction of the relationships.

### 2.3 Ethical clearance

The Ethics Committee of the Faculty of Medicine, Universitas Padjadjaran, Indonesia, has approved the study under the ethical clearance number 813/UN6.KEP/EC/2023.

## 3 Results

Table 1 presents the results of the *P. knowlesi* cross-checking examinations from 2011 to 2020. Of the 212 suspected cases referred to the national laboratory, 67 were confirmed as *P. knowlesi* infections by PCR examination. Aceh province, which referred 63 suspected cases, accounted for the highest number of confirmed infections (*n* = 31, 46.3 %) over the ten year period. Other provinces in Sumatra with confirmed *P. knowlesi* cases were West Sumatra (*n* = 5, 7.5 %), North Sumatra (*n* = 2, 3 %), and Jambi (*n* = 1, 1.5 %). In Borneo, confirmed cases were reported in South Kalimantan (*n* = 14, 20.9 %), Central Kalimantan (*n* = 8, 11.9 %), and East Kalimantan (*n* = 6, 9 %). In contrast, South Sulawesi, a province in Sulawesi, referred four suspected cases, none of which were confirmed as *P. knowlesi* infections. Similarly, four provinces in Java that did not have confirmed *P. knowlesi* cases despite referring suspected cases.

**Table 1:** Number of Thick Slide Malaria *Plasmodium knowlesi* Cases Assessed in Indonesia.

Origin (Province)	*Plasmodium knowlesi*	
	Suspect (microscopic) n (%)	Confirmed (PCR) n (%)
Aceh	63 (29.7)	31 (46.3)
North Sumatra	9 (4.2)	2 (3)
West Sumatra	7 (3.3)	5 (7.5)
Jambi	1 (0.5)	1 (1.5)
Riau	5 (2.4)	0 (0)
South Kalimantan	25 (11.8)	14 (20.9)
Central Kalimantan	11 (5.2)	8 (11.9)
East Kalimantan	12 (5.7)	6 (9)
South Sulawesi	4 (1.9)	0 (0)
East Java	33 (15.6)	0 (0)
Yogyakarta	3 (1.4)	0 (0)
Banten	1 (0.5)	0 (0)
Jakarta	38 (17.9)	0 (0)
Total	212 (100)	67 (100)

Of the 212 suspected cases, 67 were confirmed as *P. knowlesi* infections by PCR at the national reference laboratory with most were single *P. knowlesi* infections (*n* = 66, 31.1 %). One case in 2015 (0.5 %) was confirmed to be a mixed infection of *P. knowlesi* and *P. vivax*.

South Kalimantan reported *P. knowlesi* infections from 2011 to 2014, while Central Kalimantan reported a case in 2014 and five additional cases in 2015, and Jambi reported its first *P. knowlesi* infection in 2015. No confirmed cases were reported nationwide in 2016–2017. In 2018, both Central Kalimantan and East Kalimantan reported one case each. The number of cases rose again in 2019, with Central and East Kalimantan reporting one and four cases, respectively. South Kalimantan saw a significant increase, with ten cases in 2019. In the same year, North and West Sumatra provinces reported two and five cases, respectively. In 2020, East Kalimantan reported one additional case. Meanwhile, Aceh consistently exhibited a higher number of *P. knowlesi* infections, with five cases in 2018, nineteen cases in 2019, and seven cases in 2020.

Table 2 shows the demographic characteristics of malaria patients. Males are 2.23 times more likely to be at risk for malaria compared to females, and this result is statistically significant (*p*-value = 0.037, 95 % CI: 0.84–5.91). There is no significant association between non-productive (<16 years and > 60 years) and productive (16–60 years) age groups with the risk of malaria infection (*p*-value = 0.535, OR = 0.42, 95 % CI: 0.12–1.53). For occupational settings, individuals working outdoors (e.g., farmers, fishermen, forestry, and mining workers) were not significantly more protected compared to those working indoors (students, civil servants), with a *p*-value of 0.116 (OR = 0.15, 95 % CI: 0.02–1.49). The origin of the sample was found to be the most significant factor (p-value <0.001) with individuals from Kalimantan having the highest risk for malaria (OR = 3.97, 95 % CI: 2.10–7.49).

**Table 2:** PCR-Confirmed Malaria Patient Demographics in Indonesia (*n* = 147).

	*P. knowlesi* Cases		Other *Plasmodium* cases				Total	P-value*	Odds Ratio (OR)**	95 % CI of Odds Ratio (OR)**	
	*Pk*	Mix (*Pk + Pv)*	Pf	Pv	Mix (Pf + Pv)	Mix (Pf + Pv + Pm)				Lower	Upper
**Sex**											
Women	9 (13.64)	0 (0)	12 (24.49)	8 (29.63)	1 (33.33)	1 (100)	31 (21.09)	**0.037**	**2.23**	0.84	5.91
Men	57 (86.36)	1 (100)	37 (75.51)	19 (70.37)	2 (66.67)	0 (0)	116 (78.91)				
**Age**											
Non-Productive (<16 and > 60 years old)	6 (9.09)	0 (0)	2 (4.08)	3 (11.11)	0 (0)	0 (0)	11 (7.48)	0.535	0.42	0.12	1.53
Productive (16–60 years old)	60 (90.91)	1 (100)	47 (95.92)	24 (88.89)	3 (100)	1 (100)	136 (92.52)				
**Occupational Settings**											
Indoor (Students, Civil Servant)	4 (6.06)	0 (0)	1 (2.04)	0 (0)	0 (0)	0 (0)	5 (3.40)	0.116	0.15	0.02	1.49
Outdoor (Farmer, Fisherman, Forestry, Mining Workers)	62 (93.94)	1 (100)	48 (97.96)	27 (100)	3 (100)	1 (100)	142 (96.60)				
**Origin of Sample**											
Java and Sulawesi	0 (0)	0 (0)	14 (28.57)	7 (25.93)	1 (33.33)	1 (100)	23 (15.65)	**<0.001**	**3.97**	2.10	7.49
Sumatra	38 (57.58)	1 (100)	29 (59.18)	13 (48.15)	0 (0)	0 (0)	81 (55.10)				
Kalimantan	28 (42.42)	0 (0)	6 (12.24)	7 (25.93)	2 (66.67)	0 (0)	43 (29.25)				

Notes: * = Chi-Square Analysis; ** = Logistic Regression Analysis; *Pk = P. knowlesi; Pf = P. falciparum; Pv = P. vivax; Pm = P. malariae.*

## 4 Discussion

Accurate diagnosis of *P. knowlesi* malaria presents unique challenges. Although microscopic examination remains the gold standard for diagnosing malaria, it has limitations in detecting *P. knowlesi* (Ministry of Health of the Republic of Indonesia, 2020; Directorate General of Disease Prevention and Control M of H of TR of I, 2017). The morphological similarities between *P. knowlesi* and *P. falciparum* in its early trophozoite stage, and *P. malariae* in later stages, can lead to misidentification (Lee et al., 2009). This difficulty in diagnosis has been highlighted in a systematic review and meta-analysis that revealed a significantly reduced sensitivity of microscopy in detecting *P. knowlesi*, potentially contributing to misdiagnoses (Huber et al., 2023). Molecular techniques, particularly PCR, offer a more reliable method for *P. knowlesi* detection due to their ability to differentiate between *Plasmodium* species. In response to this, Indonesia has mandated the use of PCR as the diagnostic method for suspected *P. knowlesi* cases (Directorate General of Disease Prevention and Control M of H of TR of I, 2017). This requirement has driven the establishment of a national malaria laboratory network, ensuring that suspected cases are sent for PCR confirmation of *P. knowlesi* infection. (Ministry of Health of the Republic of Indonesia, 2020).

Of the 67 *P. knowlesi* cases identified*,* one mixed infection with *P. vivax* was found. Mixed infections of *P. knowlesi* with other human malaria species have been reported in several studies (Sulistyaningsih et al., 2010; Putaporntip et al., 2009; Luchavez et al., 2008; Jongwutiwes et al., 2011). One proposed mechanism for these mixed infections is transmission through mosquito bites, either from a single mosquito carrying multiple Plasmodium species or multiple mosquito bites (Ruiz Cuenca et al., 2022). This is supported by studies demonstrating the presence of mixed Plasmodium infections, including *P. knowlesi* with *P. falciparum* and *P. vivax*, in *Anopheles dirus* vector (Nakazawa et al., 2009; Maeno et al., 2015; Wong et al., 2015). Accurate identification of all Plasmodium species present in a mixed infection is crucial. Microscopic examination alone can lead to misdiagnosis in such cases. Failure to diagnose mixed infections accurately can have significant consequences, including repeated diagnostic testing and treatments, unnecessary financial burdens, inappropriate drug selection, and potentially ineffective malaria control policies (Mayxay et al., 2004).

The trends of malaria infection in Southeast Asia are indeed changing, with *P. knowlesi* infections becoming increasingly prevalent. According to the Annual Malaria Report 2022, malaria cases in Indonesia are predominantly caused by *P. falciparum* (50.55 %) and *P. vivax* (34.18 %). The remaining cases are attributed to mixed infections (13.04 %), *P. malariae* (2.07 %), *P. ovale* (0.06 %), *P. knowlesi* (0.01 %), and other species (0.09 %) (Directorate General for Disease Prevention and Control, 2023). Sumatra and Borneo are the islands in Indonesia that have reported *P. knowlesi* malaria cases (MacKinnon et al., 1997). The finding of this study highlights the region of origin as the most significant risk factor *for P. knowlesi* infection, with individuals from Kalimantan (the Indonesian part of Borneo Island) facing the highest risk (Table 2). Kalimantan is located adjacent to Sabah and Sarawak in Malaysia, where *P. knowlesi* is the dominant malaria species (Hussin et al., 2020). Borneo, the third largest island in the world, is home to vast rainforests (Indonesia, 2014), accounting for 30.3 % of Indonesia's total forest cover according to the Indonesian Geospatial Information Agency (*Badan Informasi Geospasial*, BIG) (Ahdiat, 2023). Furthermore, Kalimantan is a habitat for *Macaca fascicularis* (Moyes et al., 2016a), one of the natural hosts of *P. knowlesi*, along with *Macaca nemestrina* (George Robert Coatney, 1971). These macaques migrated to the Indonesian archipelago approximately 18,000 years ago (Fooden, 1997) and are found throughout the Southern part of the Southeast Asia, including Java, Sumatra, and Borneo in Indonesia (Mitsuru and Fooden, 2005; Roos and Zinner, 2015). *P. knowlesi* infection is transmitted through the bite of infected Anopheles mosquitoes, specifically *An. latens*, *An. leucosphyrus*, and *An. Balabacensis,* which acts as the vector that spreads the parasite from primates to humans. The widespread distribution of natural hosts and vectors on Borneo Island likely contributes to 28 (41.8 %) cases in this study (Table 1). However, due to limited evidence, the potential of human-to-human transmission cannot be ruled out and warrants further investigation.

Sumatra Island, another area with confirmed *P. knowlesi* infections, is inhabited by *M. fascicularis* and *M. nemestrina* (Moyes et al., 2016a). Interestingly, *M. nemestrina* favours areas with a low human population density and is less common in plantations (Marchal and Hill, 2009), while *M. fascicularis* exhibits a more significant presence in plantation settings (O'Brien et al., 2003). This difference in habitat preference could influence the distribution of *P. knowlesi* on the island (Moyes et al., 2016b; Ekawati et al., 2020). The presence of suitable vectors, such as *Anopheles balabacencis (*van de Straat et al., *2022**)* and *An. cracens (*Vythilingam et al., *2021**)* further contributes to *P. knowlesi* infection in Aceh. Despite a significant number of *P. knowlesi* infections reported in Sumatra between 2011 and 2020, pinpointing the precise causes and differentiating factors from Borneo island remains challenging. This difficulty stems from gaps and inconsistencies in data documentation, potentially due to limited resources or the factors hindering comprehensive information gathering. Nevertheless, this study identifies Aceh as a hotspot for *P. knowlesi* infection in Sumatra, consistent with previous systematic reviews (Bin Said et al., 2022).

Competent vectors and natural parasite hosts, like macaques, are essential for *P. knowlesi* transmission to humans (Shearer et al., 2016; Moyes et al., 2016b). Ecological disruptions, mainly deforestation, can significantly impact this delicate balance (Stark et al., 2019; Brock et al., 2019; Burkett-Cadena and Vittor, 2018; Hawkes et al., 2019). Deforestation of macaque habitats disrupts their populations and densities (Stark et al., 2019; Fornace et al., 2016) and can lead them to seek new habitats closer to human settlements (Vythilingam et al., 2014). The study shows a negative correlation between forest cover and vector-borne diseases (Morand et al., 2014), highlighting the potential consequences of deforestation. This disruption can alter vector distribution, mosquito biting behavior, and ecological adaptations (Hawkes et al., 2019), increasing the likelihood of zoonotic malaria transmission (Ekawati et al., 2020; Fornace et al., 2019a; Grigg et al., 2017). Deforestation can trigger a cascade of effects on mosquito ecology and biology, including alterations in breeding habitats, species composition, resource availability, predation patterns, and survival rates. These factors can ultimately facilitate the transmission of *P. knowlesi* from macaques to humans (Scott, 2020). Therefore, understanding the complex interplay between deforestation, vector ecology, and macaque behavior is crucial for mitigating the risk of *P. knowlesi* infections.

This study found that males are significantly more likely to be at risk of *P. knowlesi* infection than females (Table 2). This finding aligns with previous research indicating a potential link between gender and *P. knowlesi* infection risk (Grigg et al., 2017). At the same time, some studies have not shown a direct association between men and increased infection rates (Fornace et al., 2018). The higher risk observed in men in this study could be attributed to occupational exposure (Grigg et al., 2017; Fornace et al., 2018; Herdiana et al., 2016; Sato et al., 2019), as men are more likely to engage in outdoor activities like farming and plantation (Fornace et al., 2019a; Herdiana et al., 2016; Grigg et al., 2014). Previous research has consistently highlighted the association between *P. knowlesi* infection and activities such as forestry (Herdiana et al., 2018; Ekawati et al., 2020; Fornace et al., 2019a; Barber et al., 2013; Fornace et al., 2019b), agriculture (Ekawati et al., 2020; Fornace et al., 2019a; Fornace et al., 2019b) and residing in rural areas near forests (Herdiana et al., 2016; Manin et al., 2016). These activities often involve increased exposure to mosquito vectors and macaque populations. Although this study could not pinpoint the occupations with the highest risk due to data limitations, the findings underscore the importance of implementing effective malaria control measures, particularly for individuals working in outdoor settings. Integrating protective measures into occupational attire for workers in high-risk areas like plantations and agricultural lands could reduce potential *P. knowlesi* transmission (Tangena et al., 2016; Crawshaw et al., 2017).

Serological studies indicate widespread exposure to *P. knowlesi* across age groups and genders, with a higher likelihood of infection in older individuals (Fornace et al., 2018). A positive correlation has been found between seroprevalence and age for all tested antigens (Fornace et al., 2018), suggesting cumulative exposure over time. This age-related pattern is further supported by studies in Sabah (Malaysia) (Grigg et al., 2017) and Aceh (Indonesia) (Herdiana et al., 2016), which reported increased *P. knowlesi* infection rates in individuals aged 15 years and above compared to younger age groups. The higher infection risk in older individuals, mainly those aged 15–64, may be attributed to increased occupational exposure associated with this demographic (Amir et al., 2018). However, serological investigations also revealed that all age groups, including children, are vulnerable to *P. knowlesi* infection (Fornace et al., 2018). This finding is consistent with reports of *P. knowlesi* infections in juveniles without occupational exposure or travel history from Sabah, Malaysia, and Indonesia. These cases suggest that transmission can occur near or within households, highlighting the potential for peridomestic exposure to *P. knowlesi* (Herdiana et al., 2018; Grigg et al., 2017; Herdiana et al., 2016; Manin et al., 2016).

We utilized the best national data on *P. knowlesi* infection provided by the National Laboratory Network of Malaria Examinations. However, our study does not directly represent all *P. knowlesi* cases across the entire territories of the Republic of Indonesia because the laboratory has only documented cases with limited variables. Nonetheless, the data's validity should not be questioned, as certified examiners have accounted for and validated all samples. The laboratory network must comprehensively document important variables from all suspected *P. knowlesi* infection samples to enhance data quality, making data analysis more comprehensive and informative. Further research, especially qualitative studies, is crucial to understand better the risk factors associated with *P. knowlesi* infections. A high-quality epidemiological profile with comprehensive patient characterization will enhance the success of more robust and targeted public health interventions for this disease.

## 5 Conclusion

In conclusion, *P. knowlesi* malaria poses a significant public health concern in Indonesia, with cases reported in Sumatra and Kalimantan. Occupational settings and sample origin appear to be the key factors for increased risk of infection. However, accurate surveillance and reporting of *P. knowlesi* cases are hampered by resource limitations in these regions where the parasite, its vectors, and natural hosts are prevalent.

To improve diseases surveillance and implement effective control measures, a concerted effort is needed to enhance data quality related to *P. knowlesi* infections. Achieving this goal will necessitate comprehensive stakeholder engagement and substantial in resources, infrastructure, and technology.

## CRediT authorship contribution statement

**Nisa Fauziah:** Writing – review & editing, Validation, Supervision, Methodology, Data curation, Conceptualization. **Karomahul Malaya Jati:** Writing – original draft, Methodology, Formal analysis, Conceptualization. **Fedri Ruluwedrata Rinawan:** Writing – review & editing, Validation, Methodology, Data curation, Conceptualization. **Naufal Fakhri Nugraha:** Writing – original draft, Formal analysis, Conceptualization. **Bachti Alisjahbana:** Writing – review & editing, Supervision. **Jontari Hutagalung:** Writing – review & editing, Validation, Investigation, Data curation, Conceptualization.

## Declaration of competing interest

All authors confirm that they do not have any associations with or participation in any institution or entity with any monetary stake or non-monetary interest in the topic or materials discussed in this study.

## Data Availability

On reasonable request, the corresponding author can share any data supporting this study's findings.
